# Perception and Decision-Making in Virtual Telepsychology Spaces and Professionals

**DOI:** 10.3390/vision9020043

**Published:** 2025-05-06

**Authors:** Luis-Lucio Lobato Rincón, Maria Ángeles Medina Sánchez, Rubén Tovar Bordón

**Affiliations:** 1 Department of Experimental Psychology, Cognitive Processes and Speech Therapy, University Complutense of Madrid, Campus de Somosaguas, Carretera de Húmera s/n, 28223 Pozuelo de Alarcón, Spain; 2 Department of Statistics and Data Science, University Complutense of Madrid, Avda. Puerta de Hierro s/n Ciudad Universitaria, 28040 Madrid, Spain; amedina@estad.ucm.es; 3 Independent Researcher, 28600 Navalcarnero, Spain; ruben.tovar@terapiaencasa.es

**Keywords:** space perception, visual decision-making, telepsychology, environmental psychology, virtual environments

## Abstract

Humans interact with virtual environments for a variety of purposes, and the use of telemedicine services and e-health platforms has become increasingly significant in recent years. Telepsychology is an emerging service and is understood as the provision of psychological aid and counseling through the use of telecommunication technologies. This study investigates the impact of visual backgrounds in a mental health simulation app and explores the decision-making of potential patients for telepsychology. In this experimental study, we developed an instrument to assess the preferences for manipulated features of photomontages and portraits. A total of 310 participants from diverse backgrounds were surveyed about their preferences regarding visual backgrounds and silhouettes for this hypothetical service, with key independent variables such as complexity, naturalness, and color hues being manipulated. Our findings represent the first example of data collected on background preferences in telepsychology. The results suggest a preference for backgrounds with medium complexity and naturalness for clinical settings. Additionally, we observed variations in preferences based on gender and age. These findings highlight the importance of customizing visual elements to enhance patient engagement in telepsychology.

## 1. Introduction

Since its inception, clinical psychology and psychotherapy have been used in the treatment of patients in person. Currently, the advancement of technology has enabled the consultation of people with mental health issues through digital means, thanks to online videoconferencing platforms. This new form of treatment, called telepsychology, has been emerging as a highly required option, even more so after the COVID-19 pandemic, in which 67.32% of North American clinical psychologists reported carrying out all their therapeutic work via telepsychology [1]. According to the American Psychological Association [2], this service is defined as “the provision of psychological services through the use of telecommunication technologies […] by electrical, electromagnetic, electromechanical, electro-optical or electronic means” (p. 792).

Telepsychology and its most known service, videoconferencing psychology, offer the user greater accessibility to psychological assistance and certain limiting variables such as distance and mobility to the clinical center are eliminated or mitigated, thus allowing significant savings in time and money. Moreover, videoconferencing psychotherapy and other services from telepsychology favor the comfort and safety of the environment in which the therapeutic session takes place [3] and allow us to fight against some long-lasting problems in medical assistance such as mental health costs and waiting lists, thereby improving access to mental health professionals with specialized expertise [4,5].

Similarly but less commonly known is the fact that telepsychology integrates user interface navigation throughout the therapeutic process, encompassing pre-session, real-time, and post-session activities. We precisely explore this novel aspect in our study, in particular, the effect of virtual backgrounds in telepsychology applications or platforms. First, we will briefly introduce the study of real indoor space perception, followed by the knowledge achieved about this issue in virtual environments. Before the justification, we will also introduce the study of dressing and attire in impression formation.

### 1.1. Indoor Space Perception

Our study explores how the environment is perceived in the context of a simulated mental health app. So far, this context has been physical, usually in consulting rooms. Consulting rooms are physically enclosed spaces, like offices, classrooms, and meeting rooms, and all of them provide private space for the intended purpose of interaction [6]. Within those places, the human perception of location and space is fundamental to interaction [7]. As these enclosed spaces are the main places where people stay during the day, the effect of some conditions like illumination and color have been studied.

It has been demonstrated that lighting in leisure and working environments plays a significant role [8,9] and provokes an improvement of some cognitive variables such as attention and memory [10]. In low-illuminated environments, lower temperatures of color are preferred compared to in high-illuminated rooms, where higher temperatures of color are preferred [11]. Each temperature affects several personal perceptions of places including calmness, likability, and coziness [12].

Moreover, warm colors are better remembered and more attractive than other colors, and they are an important contextual clue to navigating indoors [13]. Additionally, light colors influence the appearance of interiors [14], whereas the brightest colors maximize the perceived height of ceilings [15]; some colors are preferred for certain activities, for example, studying [16]. Finally, specific colors are preferred by visitors whose intention is to stay and navigate through clinical consultation premises [17,18].

Regarding elements of the scene, familiar rooms were preferred, eliciting more positive responses and involvement with the spectator [19]. Also, vegetation has relevance in the research of indoor spaces. In general, plants displayed in a physical room seem to improve performance and mood for many people [20,21]. Furthermore, a clear cognitive effect, such as boosted sustained attention, can be observed after exposure to natural scenes for just 40 s [11]. In addition, plants and vegetation within rooms are associated with subjective wellbeing [22] and the increased perception of air quality [23].

Regarding non-physical spaces, recent research suggests that manipulating particular features of virtual environments leads to measurable changes in key responses. Variation via virtual reality has demonstrated that large windows (but not sky type) provoke a greater perception of attributes such as spaciousness in small rooms [24]. Virtual environment variation may also be achieved by online platform manipulations. By these means, non-artificially modified backgrounds were found to be preferred by students [25] and undergraduates tended to be more relaxed during work interviews when the interviewer has a natural background [26]. Also, natural backgrounds enhance creativity in Zoom meetings [27], and similarly, a view of nature from a window seems to enhance several cognitive functions during task solving [28]. Nevertheless, as far as we know, no research has studied perceptive preferences when simulating different virtual settings in e-mental health and telepsychology services.

### 1.2. The Role of Professional Appearance

On the other hand, dress and attire are very relevant features for person perception, but are under-studied [29], even more so in an emerging field such as telepsychology. In general, the perceived formality of the attire provides cues about the individual’s social status and dominance [30], and this is especially true for males [31]. Even small changes in male clothing can favor attributes such as confidence and trustworthiness [32].

Attire and appearance have been studied by several health professionals. Casual attire in physicians indicates more empathy for male patients [33], although health professionals wearing a white coat implies a higher authority and trust perception by the general population [34], as well as greater suitability and capability [35]. Regarding psychotherapy, it has been seen that physical attractiveness in female therapists favors a more comfortable client’s disclosure [36]. In addition, clients prefer moderate styles of dress for their counselor that are not too formal and not too casual [37].

Few factors have been studied in relation to a therapist’s appearance and telepsychology. For example, Pfender & Caplan [38] found that video therapists’ eye contact and gestures impact the positivity of the impression formed, with gestures having a larger effect. More research on the appearance of therapists and their attire is required, and our study seeks to fill this evidence gap.

### 1.3. Justification

The telemedicine market, which includes services for psychotherapy and mental health, is projected to exceed $590 million by 2032 [39] and one of its strongest points is being patient-centered. Any attempt to improve the closeness between the potential client and the provider of telepsychology services has its importance nowadays. Several modalities are considered for telepsychology [40,41]. A recent review [40] indicates that telepsychiatry, a field closely related to telepsychology, is the second most common service implemented on telehealth platforms that utilized virtual reality, particularly for the treatment of certain phobias. In this study, we focus on videoconferencing as the modality of telepsychology [41], which is more common for regular and broad psychological treatments.

The image design in videoconferencing telepsychology has not been extensively studied. Although technical issues are treated in telepsychology research, they typically refer to aspects tangentially related, such as suboptimal audio and visual quality [42,43], environmental distractibility [41], or the relevance of zooming in or out of the client’s expressions [44].

Considering that telemedicine and telepsychology are so relevant nowadays, poor usability or unfriendly telemedicine dispositions may hinder patient acceptance and adoption of the service [45]. Furthermore, although in general, telepsychology or online therapy is well accepted by patients, with benefits on mental health [46] and appropriate ratings of the therapeutic alliance demonstrated [47], no deep exploration of the perception of spaces for therapeutic purposes has been performed.

We are currently navigating from a world where we remain mainly within indoor spaces [48] to a world where virtual environments for working, studying, and consulting are fully available [49,50]. Although optimal designs for mental health and telemedicine apps are important nowadays [51], few studies consider the contextual cues of where patient–clinician interactions take place. The concept of global characteristics of the image [52,53] allows us to investigate certain attributes of the image, easily detected by humans and very informative, to provide a holistic description. The chosen characteristics for this study are naturalness (the degree of verticality and horizontality displayed on the image as opposed to the undulating edges more representative of nature) and roughness or complexity (the number of elements that a certain scene contains), together with color.

Our predictions are based on relevant hypotheses in the area of perception and behavior. First, we argue that naturalness in the viewed scene will be preferred for mental health consultation by our participants over other spaces. This is based on biophilia hypothesis. Biophilia is “an unconscious and innate need to affiliate with nature and living organisms” [54] (p. 792). In a study about automatic associations, people were faster when choosing a natural environment than urban settings [55]. Although not all people seem to have such an attraction for nature, most of us show a specific pattern of autonomous activity when observing natural landscapes [56] and these are often preferred for certain work tasks [57].

The variable complexity is also studied in this manuscript. This characteristic can be explained from an evolutionary viewpoint. Kaplan [58] defended that humans, as with other animals, could have preference for information to fill up their cognitive maps (or mental models as Johnson-Laird [59] conceptualizes). The processes behind these preferences would be unconscious and dominated by an adaptive function similar to choosing a habitat [60]. In landscape, complexity has been found to have an impact on a preference for open landscapes and forests [61,62]. When some artificial element is present in the scene, complexity is a main factor that contributes to preference [63], and complex interiors are also preferred over simpler indoor spaces [64].

## 2. Materials and Methods

### 2.1. Design and Ethical Approval

In this experimental design, all the participants selected their preferred environments (or male/female figures), and the dependent variables were the proper selection and the response time. The independent variables were the manipulation of complexity and naturalness within these environments.

Ethical approval was obtained from the Complutense University Institutional Review Board (Id number: CE_20220217-06_SOC) and the questionnaires were administered from 1 January to 31 December 2023.

### 2.2. Sample

We recruited 310 people over 18 years old (M = 39.06; SD = 13.7) in a convenience sampling where 59.7% were women. The pervasiveness of the knowledge and use of health applications allowed us to recruit the participants with minimum constraints. We recruited the participants from the personal and professional networks of the authors and collaborators, mainly from www.terapiaencasa.es URL (accessed on 29 April 2025), an online psychological consultation service in Spain. The only inclusion criteria were to be an adult over 18 years old and to possess a device to access the survey research, and the exclusion criteria were to have any visual deficit.

### 2.3. Images Creation

For our study, human figures and indoor spaces were designed by two professional graphic designers.

**Figure 1:**
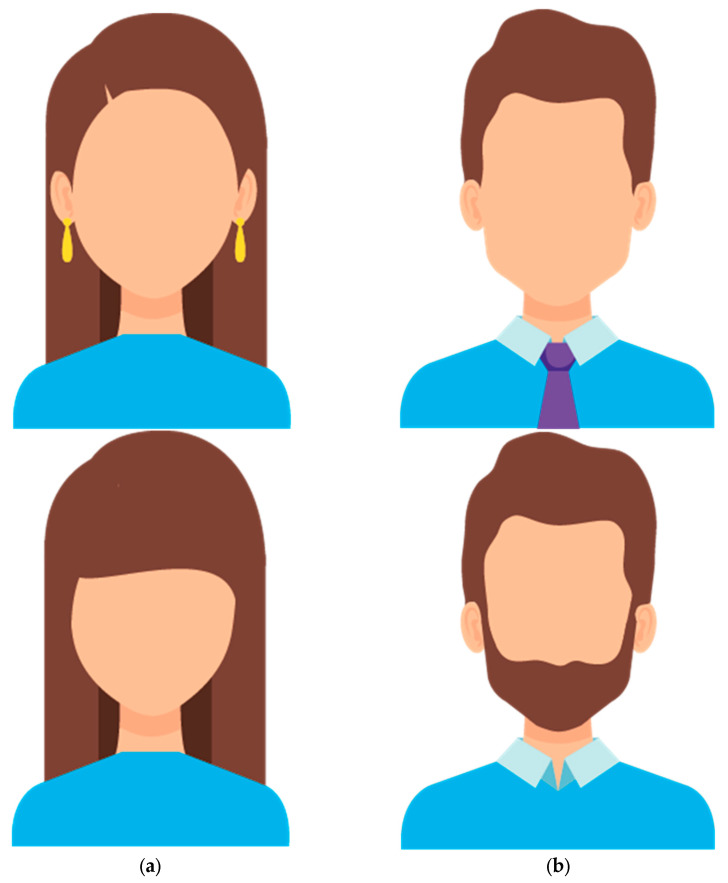
(**a**) Two options for the female therapist (dressed-up vs. casual attire); (**b**) two options for the male therapist (dressed-up vs. casual attire).

Designs with human figures were initially selected on the bank image Freepik (www.freepik.es URL (accessed on 29 April 2025)). This selection was followed by precise modifications performed by the software Adobe Illustrator CS6 (Adobe, 2012). Human figures were edited with the pen tool simplifying facial traits and with the color picker option to include solid colors. The results of these operations were the final human figures for this study. Experimental manipulation in this case was created by modifying the extension of hair in both face silhouettes: females and males. The images corresponded after the modification with an increase in low frequencies. The other option in both cases was the reduction in the hair area and the inclusion of one complement, thus introducing details. This way, for the other pair of images, there was an increase in high frequencies. These variations can be seen in Figure 1.

Regarding photomontages of indoor images, some examples were searched on the internet with Google motor research. Both the background and details for the final photomontages were combined in congruent tone and perspective. The image-editing software Photoshop CS6 (Adobe, 2012) was also used for this task.

**Figure 2:**
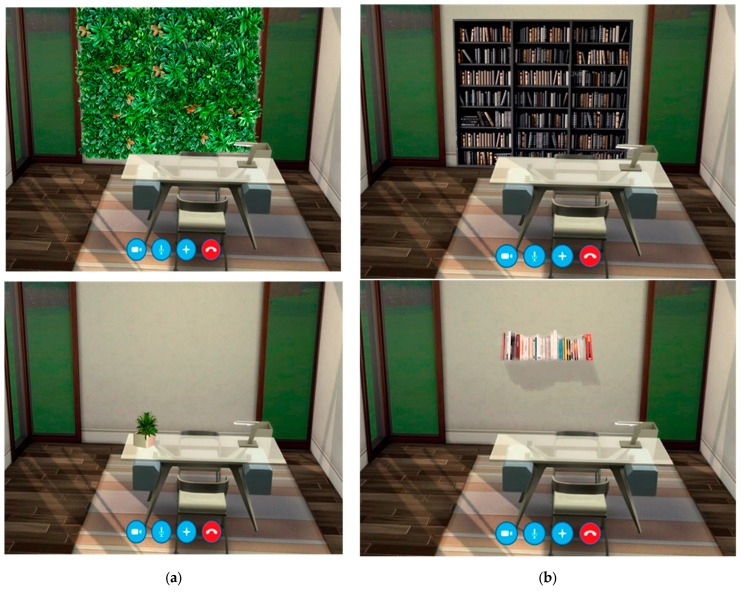
(**a**) High vs. low naturalness in telepsychology environment; (**b**) high vs. low complexity in telepsychology environment.

As it has been mentioned, we chose preferentially two global characteristics of the image to manipulate: complexity and naturalness [53]. Naturalness (Figure 2a) was manipulated with the inclusion of mainly indoor vegetation and plants, and complexity (Figure 2b) was manipulated increasing the number of elements present in the room [64]. Similar natural and artificial indoor environments have been manipulated in previous studies [57,65].

**Figure 3:**
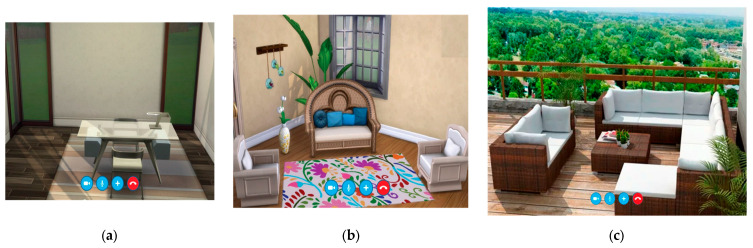
(**a**) Low level of both complexity and naturalness for telepsychology environment; (**b**) medium level of both complexity and naturalness for telepsychology environment; (**c**) high level of both com-plexity and naturalness for telepsychology environment.

A third manipulation for photomontages consisted of the mix of these two attributes, where the first image was a plain office, the second included some plants and decoration, and the last one was an open environment with wood-made coaches and a view of nature. These mixed backgrounds can be viewed in Figure 3.

Focusing on color environments, we also asked for the chromatic preferred option for the ambience in hypothetical telepsychology interactions. Nine colors were selected between those proposed by [17] for counseling rooms. In Table 1, the link between the Hex code, RGB code, and name for the colors surveyed is shown.

### 2.4. Instrument

The implementation of the online questionnaire was carried out using a combination of HTML5 (Hypertext Markup Language 5), CSS (Cascading Style Sheets, version 3), JavaScript (ES5), and Firebase (version 7.14.2.). HTML was used to define the structure of the questionnaire, CSS to design the visual appearance, and JavaScript to add interactivity and dynamism.

The database was hosted and managed on Firebase. The connection of the database with the web page is carried out by the Firebase API, which allows for the extraction of the data entered in the questionnaire for its subsequent analysis and evaluation. Using cookie recognition on the user devices, we prevented any user from answering the questionnaire more than once from the same device. In addition, the user had to provide an alphanumeric code provided by the research team to avoid misuse of the questionnaire. Together, these security measures guaranteed the integrity and confidentiality of its data, in addition to maintaining the quality of the responses obtained.

As our study focused on decisions based on perceptual cues, the study needed fast and automatic responses, not caused by too much rationalization, as [66] have suggested. Therefore, response time was free for participants, although later on, only responses below 20 s were selected for the analysis.

### 2.5. Procedure

**Figure 4:**
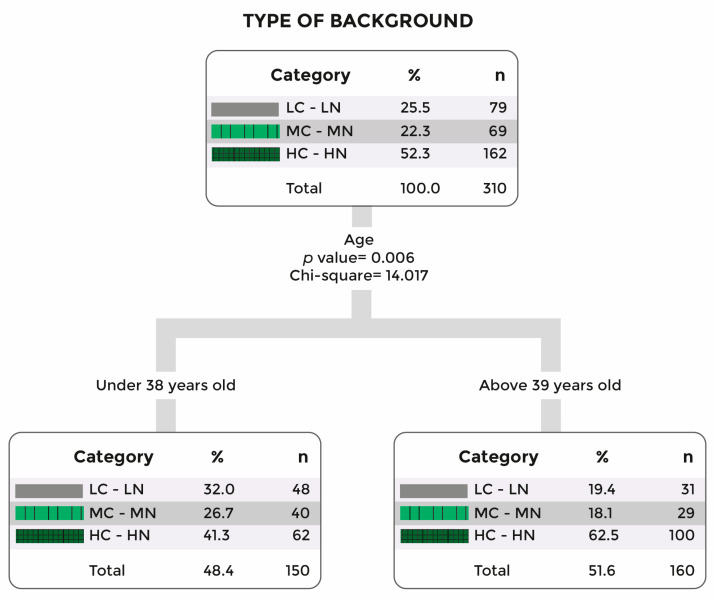
Decision tree for the responses to the question: “Which of the following environments would you prefer if you needed online therapy?”. Note: LC-LN for Low Complexity and Naturalness; MC-MN for Medium Complexity and Naturalness; and HC-HN for High Complexity and Naturalness.

Participants showed their willingness to participate in the research project and then contacted the main researcher, LLLR. A particular code was linked to their set of responses in order to treat their responses anonymously. The first part of the online survey was about sociodemographic data with multiple-choice questions about the use and interest in psychological therapy, among others. The second part was about visual preferences. The questions displayed in this part were as follows. (1) Preference about the therapist’s sex; (2) preference about the preferred color for telepsychology interaction (showing the hues codified in Table 1); (3) “Which of the following environments would you prefer if you needed online therapy?” (Figure 3); (4) “What (female) therapist appearance do you prefer in case you should choose one in a mental health app?”; (5) “What (male) therapist appearance do you prefer in case you should choose one in a mental health app?” (both in Figure 1); and (6) and (7) “Between the two options, what type of decoration would you feel most comfortable with if you needed online psychological therapy?”, with the final question being formulated for both cases: naturalness (Figure 2a) and complexity (Figure 2b).

The questionnaire was completed, on average, in 4 min. All the reaction times for each preference response were measured, together with the option chosen.

After the completion, participants were paid 1 euro by the Spanish payment service provider Bizum.

### 2.6. Data Analysis

The statistical analysis followed to achieve the objectives presented was as follows:

- Analyzing the ANOVA for time responses and chi-square tables for categorical variables to see what factors influenced the user when choosing an online environment or taking any option.
- Grouping individuals for each photomontage through decision trees to take their profile and characteristics into consideration.
- Finally, calculating the probability that a given user uses the different proposed environments thanks to logistic regression models.

The analysis was carried out with the SPSS statistical package (IBM, version 27).

## 3. Results

### 3.1. Descriptive Statistics

The sample mainly responded through their mobile phones (88.7%) and 156 participants (50.3%) had received psychological therapy previously. We also asked for the level of education and the level of interest in psychological therapy, which can be seen in Table 2. In this study, most people surveyed have completed university studies (65.2%) and had a clear interest in receiving psychological therapy in the near future (81.7%).

When facing the demand about the preferred gender for the counselor in the telepsychology service, people opted mostly for “Indifferent” and a very low percentage (ranging from 2.6% to 13.6%) preferred a male psychologist within all ages. Even for men, the option of the male psychotherapist was the least demanded (16%).

### 3.2. Therapist Appearance

In general, participants had no preferences over therapist gender and the second most popular choice was a female therapist over a male therapist (χ^2^(6, *N* = 310) =20.71, *p* ≤ 0.01). When selecting potential therapists according to their attire, younger participants consistently chose the formally dressed female figure over other options (χ^2^(3, *N* = 310) = 16.70, *p* ≤ 0.01). This preference for formal attire was also observed in their selection of male therapists. Conversely, participants over 50 years old preferred casually dressed male therapists, unlike the younger group’s choice (χ^2^(3, *N* = 310) = 23.50, *p* ≤ 0.01).

In addition, an analysis of response times revealed a significant difference in how quickly participants chose the male therapist’s appearance. Specifically, participants who expressed ‘indifference’ towards future psychological treatment took significantly longer to make their choice compared to those who had clear opinions about having either no interest or a high level of interest in therapy (F(4, 277) = 2.715, *p* = 0.03). However, this delay in decision-making among indifferent participants was not observed when they were choosing the female therapist’s appearance.

On the contrary, when an opinion on the appearance of a male therapist was requested, the age of the participants did not influence the time taken to respond, but it was an influence when they were asked about the female therapist’s appearance (F(3, 278) = 2.817, *p* = 0.04). In fact, those under 39 were faster when deciding between the two female figures than those above 39.

### 3.3. Decision-Making for Virtual Environments in Telepsychology

Our study also analyzed how visual cues in a virtual environment influence decision-making among potential telepsychology users. Regarding the question, “Which of the following environments would you prefer if you needed online therapy?”, we must remember that participants had three options. The simpler option had no details, and the complexity and naturalness increased in the next two options progressively, with a moderate number and maximum level of details and plants in the third option.

Participants over 39 preferred backgrounds with medium complexity and naturalness (χ^2^(6, *N* = 310) = 16.66, *p* = 0.01). Younger participants showed no strong preference, though they also most frequently chose the medium complexity/naturalness background. The decision tree for this question also showed a clear separation between the marked preference of those older than 39 to those younger (see Figure 4). Regarding time responses, the medium configuration of complexity and naturalness (illustrated in Figure 3b) provoked the fastest response with a mean of 1.5 s, followed by the simpler room configuration as the second faster (F(2, 291) = 4.10, *p* = 0.02).

The chi-square contrasts revealed no statistically significant differences between genders or based on previous experience with therapy regarding the selection of any virtual environment.

Regarding the type of decoration (Figure 2a,b), when a decision between opposite levels of complexity (or naturalness) was requested, all groups of ages preferred backgrounds with higher complexity and naturalness (χ^2^(6, *N* = 310) = 16.66, *p* = 0.01).

**Figure 5:**
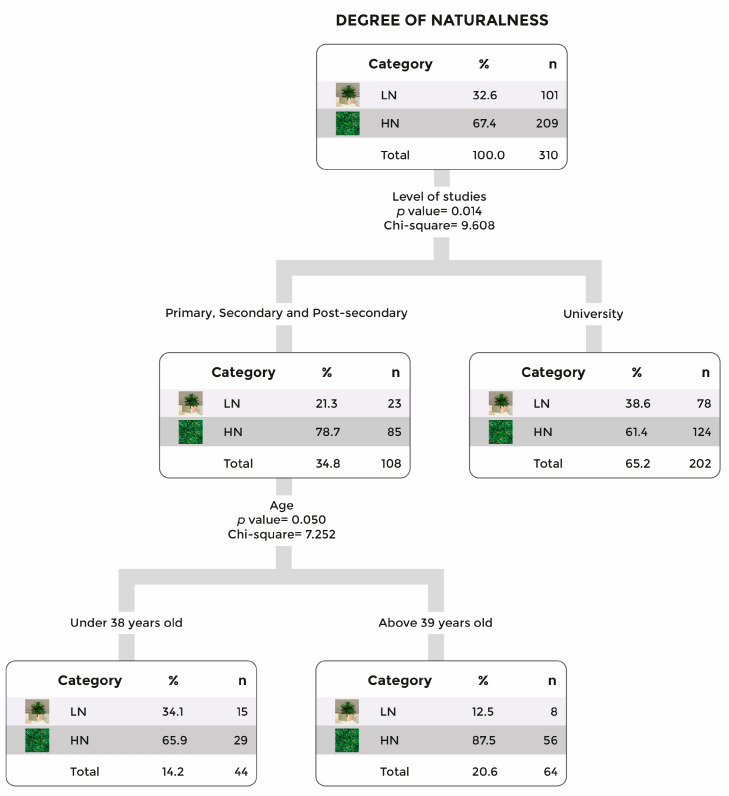
Decision tree for the responses to the question: “Between the two options, what type of decoration would you feel most comfortable with if you needed online psychological therapy?”. Note: LN for low naturalness and HN for high naturalness.

Specifically analyzing responses regarding the preferred level of naturalness in the images, a decision tree was constructed to group participants based on their responses. In this tree that is shown in Figure 5, a higher percentage of participants with primary, secondary, and post-secondary education selected the natural scene compared to those with university education (78.7% vs. 61.4%). As shown in Figure 5, older participants without higher education displayed the strongest preference for this scene.

On the contrary, the question of furniture decoration according to the complexity or number of details did not allow us to group participants according to a clear profile as the natural decoration did.

Regarding the preferred colors for the atmosphere of the telepsychology service, the cerulean option, a bluish color, was the most popular (38.7%), followed by observatory (19.7%), a color with greener hues. Cerulean was selected more by females (44.3% vs. 30.4%), whereas observatory was selected more by males (14.6% vs. 27.2%). These differences are significant (χ^2^(8, *N* = 310) = 25.97, *p* < 0.01). Lastly, taking the entire sample together, the least accepted hues for telepsychology videoconferencing were reddish hues like Tia María style (2.3%) and cabaret style (2.9%).

### 3.4. Logistic Regression for Choices

When we applied logistic regression to the questions directed at both preferences over environments and over decorations, the variable(s) directly related to each choice were taken into account. Specifically, for the question “Which of the following environments would you prefer if you needed online therapy?” (see Figure 3a–c), the related variable was age (χ^2^(6, *N* = 310) = 13.96, *p* = 0.03), so it was included in the model. Taking the metallic environment category (gray counseling room) as a reference, it was found that those aged over 39 had at least twice the probability of choosing the medium combination between naturalness and complexity background (OR = 2.6; *p* = 0.1 for those between 39 and 50 years and OR = 3.8; *p* < 0.01 for those above 50 years old), compared to younger participants.

Regarding the questions about decorations with natural motifs (vertical garden vs. potted plant) and furniture (full book shelf vs. shelf), no variable was significant to calculate the probability of choosing these decorations, so the logistic regressions did not show relevant results. Finally, in the case of plant decoration, a tendency could be observed for those over 39 years of age with non-tertiary studies (vocational training, primary, and secondary) to choose options with high nature to a greater extent. This can also be seen in the decision tree for the question, as has been previously presented (Figure 5).

## 4. Discussion

A growing demand for healthcare apps and software design prompted us to conduct the present work. In our study, we recruited participants as potential patients of e-mental health services to analyze how their decision-making about key elements in therapy could vary according to their visual impression. There were three main objectives: to analyze responses according to the appearance of the therapist, to analyze responses according to global characteristics of the image, and to predict responses from specific profiles of participants.

### 4.1. According to Appearance of the Therapist

In the realm of person perception, something interesting to consider is that “context” encompasses a broad range of visual aspects of the environment that provide a source of expectations and predictions about the social targets likely to be perceived in that environment. The cues manipulated in this study were the style or look in both cases for female and male therapists.

An interesting duality can be seen in our study, which breaks the general rule that, in professional contexts, someone dressed formally receives more positive evaluations and favoritism than people with casual or less stylish attire [32,67,68]. In our case, participants older than 50 preferred well-dressed male counselors, but casual-looking women were selected when the decision was between female faces. On the contrary, young people selected the inverse pattern, dressed-up female therapists vs. casually dressed male therapists. This preference for professional women dressed in formal attire aligns with findings by [69] who reported that young people perceived female violinists in concert dress as more technically proficient and competent than those in nightclub attire. This element adds to the knowledge that women are more likely judged by visual aspects by young people [70].

The preference for casually dressed male therapists might be explained by previous findings [71] suggesting that attire is not the primary factor influencing perceptions of competence in men. Furthermore, the beard, the element used in this study to “represent a casual style”, could also be interpreted as a cue for intelligence and professionalism, which is very congruent with the proposal for a therapeutic intervention. In fact, a perceived smart attire positively influences the impression men make on others [72].

Also, with regards to this decision and assuming the time response as an index of the strength of preferences [73,74], we can affirm that young people have strong preferences for dressed-up female professionals, not only in terms of percentage but in terms of strength of attitude, because participants younger than 39 were faster when deciding their opinions about female appearance. Another finding coherent with the correlation between the time taken to respond and the strength of the attitude is seen when respondents who are indifferent to psychological therapy decide about appearances in a slower manner compared to those very interested.

### 4.2. According to Environmental Images

Little is known about the image characteristics that have relevance when navigating virtual environments associated with telepsychology. As we have mentioned, the global characteristics of the images studied here have been complexity, naturalness, and color. They are not processed consciously but they do influence human behavior [52,53].

In general, our results allow us to state that people prefer a virtual environment with a moderate range of items and plants and, in the case of facing a decision between spaces for telepsychology services with high or low complexity (or high or low naturalness), people generally opt for high levels of both.

Regarding complexity, medium levels of organized complexity are preferred when several mountain and hilly landscapes are presented [63,75] and this is also demonstrated in interior preferences [64]. Also, a certain level of complexity is seen when people choose the best option for backgrounds in a physician consultation [76]. So, the data are unidirectional in showing this trend, as has now been confirmed for telepsychology.

Regarding naturalness, our results showing preferences tending towards more vegetation in the background also fit with several findings. First, observers prefer architectures with a curvilinear component and this trend seems to be universal [77]. We must remember that natural scenes contain more curvilinear elements than other scenes with handmade objects, so this preference is expected according to the geometry, but also according to the vegetation itself, which has an evolutionary meaning as proposed by biophilia [54]. Also, hybrid studies combining real and computer-manipulated environments show that the presentation of indoor plants (but not windows) seems to improve the perception of certain premises [65].

This preference for vegetation even for high levels was pronounced in our study in participants over 39 who preferred the open-air photomontage (Figure 3c). Research also shows this preference for natural outdoor places when activities involve creativity such as brainstorming, reflection, and evaluation [57]. In addition, the latter study indicates that natural spaces were preferred in activities where silence is preferred (reflection, lectures, reading, etc.). It seems that this would be a stronger need for our older and middle-aged individuals, who preferred the outdoor terrace for psychotherapy sessions in this study.

However, our results reveal a preference for a moderate combination of items and plants rather than a high level of naturalness and complexity (see Figure 3b vs. Figure 3c). This preference, now observed in the context of telepsychology, aligns with findings in studies focused on productivity. Specifically, whereas plants in the office might seem to provoke a positive effect on productivity and mood, it is also true that some studies obtain these findings regarding productivity when a moderate (instead of a high) quantity of plants in the room is applied [20]. The preferred plants are especially leafy [21], as in our vertical garden in Figure 2a, and rounded [22], as in the plant displayed in Figure 3b.

The last global characteristic of the images, color, has some interest too. Color environment impact has an immense amount of literature on the topic: the influence on more fixations in landscapes [61] compared to the effects on attributes such as more useful, spacious, and clear spaces when white and green lights are the source of illumination in the room [14].

According to preferences in environmental colors, blue and green were found the most preferred colors for face-to-face mental health consultation facilities [17]. In our case, using the same palette of colors as in the former study, we found that bluish and greenish colors were the best accepted for videoconferencing psychology. In our case, we found a difference depending on sex. Males preferred a greener hue, compared to females, who preferred a bluish hue. Reddish tones were the last choice for telepsychological services by everybody.

### 4.3. Limitations and Prospective

Among the design limitations, this study is focused on the influence of the characteristics of the global image on decision-making, but we cannot disregard the possibility of the impact of the reflective processes here, which is not dependent on perception, given the extended response time allowed. However, we minimized the influence of extensive analysis and rationalization driven by Type 2 reasoning processes, as described by Evans [78,79], by limiting our analysis to responses under 20 s. Nevertheless, some degree of rationalization, beyond purely perceptual processing, remains possible among our participants.

In addition, a limitation and potential weakness of our study is that we did not analyze the link between perception and action. Hypothetical preferences from the users could not be the most demanded option in the long term because of practical issues or a combination of conscious and automatic processes. For example, some elements could be preferred but also produce distraction; likewise, intense lighting increases male participants’ memory and color affects female participants’ attention in virtual reality environments [10]. At the same time, it is possible that positive impressions of backgrounds threaten the retention of clinical data [80] and this derives in a lesser quality of the therapy. These are aspects to consider when designing and carrying out telepsychology studies and treatments.

A further limitation to contemplate in our study is the convenience sampling used to constitute the study sample. This strategy could have affected the generalizability of the findings, and therefore, results must be used with caution. Future studies should incorporate more diverse and randomized sampling approaches to enhance external validity.

Moreover, further research on telepsychology services could explore dynamic facial cues in ongoing therapeutic interviews or sessions in order to go beyond the static cues presented in this study. In addition, the use of eye gaze methodology in future studies may be paramount to determine the content where the look fixates depending on different therapists, environments, or backgrounds. Apart from the perceptual perspective, it could be very useful to incorporate the study of potentially relevant slower and reflective choices in future analyses, as it is not included here.

In general, we expect that customizing the sensory experience of consultants is a mechanism that likely drives to engagement and collaboration with therapists and professionals, enhancing physical and psychological recovery.

## 5. Conclusions

This study enhances the knowledge about the interaction between two basic human processes, perception and decision-making, analyzing the impact of visual cues on user preferences in a simulated context of telepsychology. So far, the use of visual modifications of both therapists’ and environments’ appearance has received limited exploration within telepsychology. In particular, our study reveals subtle differences in preferences regarding therapists between individuals older and younger than 50. We identified a specific influence of age on preferences in therapist appearance, with younger individuals (under 39) preferring more formal attire for female therapists. In terms of complexity and naturalness, our data support the well-established notion that moderate levels of complexity and naturalness are preferred. Another significant interaction was observed between gender and color preferences, with women favoring bluish tones and men leaning towards greener hues, according to our survey. These findings suggest potential segmentation strategies for marketing in telemedicine and telepsychology services, enabling more tailored promotional approaches and resulting in more patient adherence.

## Figures and Tables

**Table 1:** Hex, RGB codes, and color names.

HEX (RGB)	Color Name
0094de rgb (0, 148, 222)	Cerulean
#ae47e0 rgb (174, 71, 224)	Medium purple
#777777 rgb (119, 119, 119)	Boulder
#007cde rgb (0, 124, 222)	Lochmara
#028e74 rgb (2, 142, 116)	Observatory
#028d00 rgb (2, 141, 0)	Japanese laurel
#8a7500 rgb (138, 117, 0)	Olive
#d73c0c rgb (215, 60, 12)	Tia maria
#ce3e79 rgb (206, 62, 121)	Cabaret

**Table 2:** Sociodemographic and user variables of interest.

	Primary	Secondary	Post-Secondary	University	
Studies *n* (%)	17 (5.5%)	31 (10%)	60 (19.4%)	202 (65.2%)	
	**None**	**Low**	**Indifferent**	**Some**	**High**
Interest in psychological therapy	15 (4.8%)	11 (3.5%)	31 (10%)	131 (42.3%)	122 (39.4%)

## Data Availability

The raw data supporting the conclusions of this article will be made available by the authors on request.
